# Comparative genomics identifies male accessory gland proteins in five *Glossina* species

**DOI:** 10.12688/wellcomeopenres.12445.2

**Published:** 2017-11-22

**Authors:** Muna F. Abry, Kelvin M. Kimenyi, Daniel Masiga, Benard W. Kulohoma

**Affiliations:** 1 Center for Biotechnology and Bioinformatics, University of Nairobi, P.O. Box 30197, Nairobi, 00100, Kenya; 2 International Centre for Insect Physiology and Ecology, P.O. Box 30772, Nairobi, 00100, Kenya

**Keywords:** Accessory gland proteins, tsetse fly, reproductive cycle, vector control, trypanosomiasis

## Abstract

Accessory gland proteins (ACPs) are important reproductive proteins produced by the male accessory glands (MAGs) of most insect species. These proteins are essential for male insect fertility, and are transferred alongside semen to females during copulation. ACPs are poorly characterized in *Glossina* species (tsetse fly), the principal vector of the parasite that causes life-threatening Human African Trypanosomiasis and Animal trypanosomiasis in endemic regions in Africa. The tsetse fly has a peculiar reproductive cycle because of the absence of oviposition. Females mate once and store sperm in a spermathecal, and produce a single fully developed larva at a time that pupates within minutes of exiting their uterus. This slow reproductive cycle, compared to other insects, significantly restricts reproduction to only 3 to 6 larvae per female lifespan. This unique reproductive cycle is an attractive vector control strategy entry point. We exploit comparative genomics approaches to explore the diversity of ACPs in the recently available whole genome sequence data from five tsetse fly species ( *Glossina morsitans, G. austeni, G. brevipalpis, G. pallidipes *and *G. fuscipes*). We used previously described ACPs in *Drosophila melanogaster* and *Anopheles gambiae* as reference sequences. We identified 36, 27, 31, 29 and 33 diverse ACP orthologous genes in *G. austeni, G. brevipalpis, G. fuscipes, G. pallidipes *and *G. morsitans* genomes respectively, which we classified into 21 functional classes. Our findings provide genetic evidence of MAG proteins in five recently sequenced *Glossina *genomes. It highlights new avenues for molecular studies that evaluate potential field control strategies of these important vectors of human and animal disease.

## Introduction

Accessory gland proteins (ACPs) are important reproductive proteins produced by the male accessory glands (MAGs) of most insect species. These proteins are essential for male insect fertility, and are transferred alongside semen to females during copulation ^1^. ACPs trigger significant physiological and behavioral changes in females after copulation, which include: egg laying, reduced sexual receptivity and refractoriness to subsequent inseminations, induce the expression of immune peptides and reduction of female lifespan ^1– 5^. ACPs are only resynthesized after transfer of seminal fluid to females, but topical application of juvenile hormone on the male’s cuticles stimulates *in vivo* re-synthesis to pre-mating levels ^2^. Female *Anopheles gambiae* mosquitoes copulated by males with degenerate testes and MAGs fail to oviposit and readily re-mate ^1^. Conversely, those copulated by males with degenerate testes but fully developed MAGs lay unfertilized eggs and do not re-mate ^1^. This underscores the relevance of ACPs as an entry point for vector borne disease control.

ACPs are poorly characterized in *Glossina* (tsetse fly), compared to *Drosophila* and *Anopheles* species ^1, 6^. The tsetse fly is the principal vector of the parasite that causes life-threatening human (sleeping sickness) and cattle (nagana) trypanosomiasis in endemic regions in Africa ^7^. Over 60 million people and 80 million cattle are at risk of contracting disease ^8^. Female tsetse flies only mate once during their lifespan and store the male ejaculate in their spermathecae, which they subsequently use to self-fertilize ^9^. They have a peculiar reproductive cycle because of the absence of oviposition, with females producing a single fully developed larva at a time that pupates within minutes of exiting their uterus. This slow reproductive cycle, compared to other insects, significantly restricts reproduction to only 3 to 6 larvae per female lifespan ^10^. This unique reproductive cycle is an attractive vector control target. An improved understanding of tsetse fly’s reproductive biology, and specifically ACPs that are crucial determinants of successful reproduction in other insect species, may provide valuable possible vector control strategy entry points.

Comparisons between ACP gene orthologs in *Drosophila simulans* and *D. melanogaster* show they are rapidly evolving, relative to non-ACP genes ^11– 13^. However, there is strong ACP peptide structural homology between closely related species, which decreases as species phylogenetic distances increase ^14^. This rapid rate of ACP genes evolution has made it challenging to reliably identify orthologs across insect species in the absence of genomic data ^15, 16^. The recently available whole genome sequence data from five tsetse fly species ( *Glossina morsitans*, *G. austeni*, *G. brevipalpis*, *G. pallidipes* and *G. fuscipes*) has made it possible to revisit detailed examination of ACP gene distribution and genetic diversity in tsetse flies. We exploit comparative genomics approaches to interrogate these genomes, using previously described ACPs in *D. melanogaster* and *A. gambiae* as reference sequences.

## Methods

### Identification of *Glossina, Anopheles* and *Drosophila* species ACP homologs

The proteomes of *G. austeni, G. morsitans, G. pallidipes, G. fuscipes* and *G. brevipalpis* were retrieved manually from VectorBase ( www.vectorbase.org). The retrieved *Glossina* protein sequences alongside previously described ACP sequences from *A. gambiae* (n=57) and *D. melanogaster* (n=173) ( Supplementary Table 1) ^1^, used as references, were assigned to homologous clusters using OrthoMCL with default settings (BLASTP E-value cut-off 1e-5 and inflation index 2.5) ^17^ ( Supplementary File 1). Clusters with singletons were omitted from further processing. Mapped orthologs were subsequently processed using BMX ^18^.

### Sequence alignment and phylogeny reconstruction

Multiple sequence alignments were performed using MUSCLE ^19^. Maximum likelihood (ML) phylogenetic analysis of the multiple aligned sequences with bootstrap values of 100 replicates was performed using PHYML version 3.5 ^20^.

### Determining the direction and extent of selection pressure

The magnitude and direction of selection pressure on the ACP sequences was tested based on the ratio (ω = d _N_/d _S_) of the average number of non-synonymous substitutions per non-synonymous site (d _N_) to the average number of synonymous substitutions per synonymous site (d _S_). If ω = 1, amino acid substitution is assumed to be under neutral selection, ω > 1 is indicative of positive selection whereas ω < 1 is evidence of negative or purifying selection. Sequence alignments of each of the ACP clusters containing *A. gambiae*, *D. melanogaster*, *G. austeni*, *G. brevipalpis*, *G. fuscipes*, *G. morsitans* and *G. pallidipes* were generated. Each alignment was then uploaded to the SNAP program ^21^ ( www.hiv.lanl.gov), which calculates synonymous and non-synonymous substitution rates to determine the magnitude of selection pressure.

### Data visualization

A list of the relative abundance of ACPs with secretory signals identified within each ortholog cluster for *Glossina, Anopheles* and *Drosophila* species was generated ( Supplementary Figure 1). The 21 ACPs clusters were visually presented in a single circular ideogram using CIRCOS software ^22^.

## Results

### Identification of ACP gene orthologs in *Glossina* species

**Figure 1.**
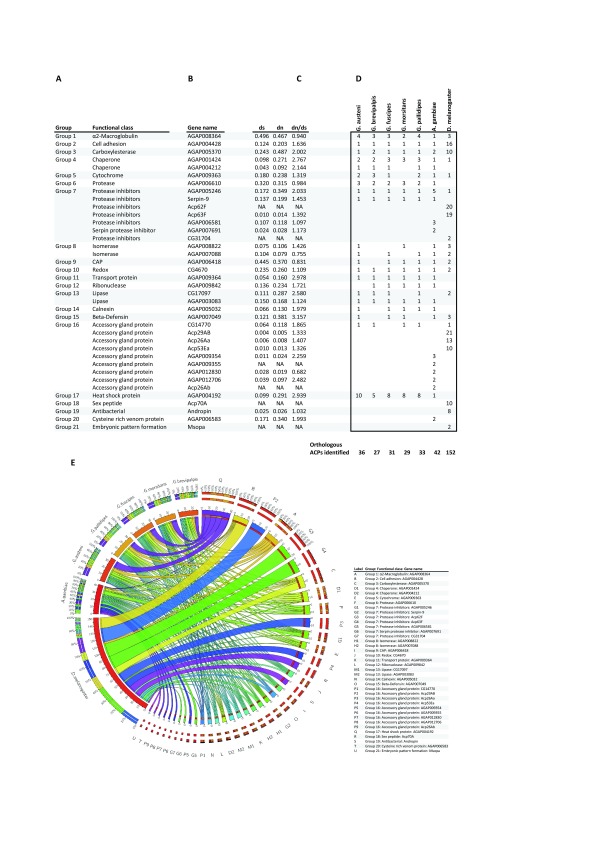
Identification of male accessory gland proteins (ACPs) in five *Glossina* species. ( **A**) We identified 38 clusters of ACPs, which were classified into 21 functional classes. ( **B**) The identified *Glossina* species ACPs are orthologous to well-characterized ACP genes in *Anopheles gambiae* and *Drosophila melanogaster genomes*. ( **C**) The magnitude and direction of selection pressure on the ACP sequences was tested based on the ratio (ω = d _N_/d _S_) of the average number of non-synonymous substitutions per non-synonymous site (d _N_) to the average number of synonymous substitutions per synonymous site (d _S_). A ratio greater than 1 indicates positive selection, and a ratio less than 1 indicates purifying selection. ( **D**) The relative abundance of ACPs identified per ortholog cluster. We identified 36, 27, 31, 29 and 33 ACP orthologs with secretory signals in *G. austeni*, *G. brevipalpis*, *G. fuscipes, G. pallidipes* and *Glossina morsitans* genomes, respectively. ( **E**) Schematic presentation showing the relative abundance of each ACP functional class in *Glossina* species, *A. gambiae*, and *Drosophila melanogaster*. α2-macroglobulins [A] and, heat shock proteins [Q] are the most abundant ACPs in *Glossina* species.

We analyzed five recently sequenced *Glossina* genomes: *Glossina morsitans*, *G. austeni*, *G. brevipalpis*, *G. pallidipes* and *G. fuscipes*, to examine the presence of orthologs to ACP genes previously identified in *Drosophila melanogaster* (n=173) and *Anopheles gambiae* (n=57) ^1, 6^. First, we identified 41 ACP ortholog clusters that we broadly classified into 23 groups based on the encoded protein’s functional class. These ACPs have a distinct species distribution with: 12 ortholog clusters common to *Glossina, A. gambiae,* and *D. melanogaster* species; and some clusters only present in *A. gambiae* and *Glossina* species (n=7) or *D. melanogaster* and *Glossina* species (n=5) ( Supplementary Table 1). The remaining 17 clusters consist of ACP orthologs exclusive to either *A. gambiae* (n=7) or *D. melanogaster* (n=10). Next, we shortlisted genes that encode proteins carrying classical secretory signals ^23^ to distinguish the matched testes-specific secreted male accessory gland proteins from other insect peptides. We identified 36, 27, 31, 29 and 33 ACP orthologs with secretory signals in *G. austeni*, *G. brevipalpis*, *G. fuscipes, G. pallidipes* and *Glossina morsitans* genomes respectively ( Figure 1), across 21 functional class groups. α2-macroglobulins (Group 1) and heat shock proteins (Group 17) are the most abundant ACP orthologs in *Glossina* species ( Figure 1). Interestingly, *Glossina* species lack orthologs to Acp70A (Group 18) and andropin (Group 19), which has antimicrobial properties and safeguards the male ejaculate, and stimulation of long-term post mating responses in females respectively ^1, 24, 25^.

### Most ACP genes are under positive selection

We inferred the direction and magnitude of selection pressure on the identified ACP orthologs using dN/dS ratios. We observed possible signatures of positive selection in all genes except five are evolving under positive selection ( Figure 1). We found that α2-macroglobulins, which have been shown to be important in mosquito and *Drosophila* immunity ^26, 27^, display signatures of purifying selection suggesting they are critical for successful reproduction and all deleterious variations are purged. Our analysis was restricted to reference genes present in *A. gambiae* and *D. melanogaster*, and future studies that integrate data from more closely related taxa will highlight evolutionary changes associated with ACPs in more detail. We reconstructed the phylogeny of ACP orthologs within each cluster ( Supplementary Figure 1). We failed to identify any pattern associated with the diverse ecological niche and unique reproductive style in *Glossina* species in our analysis.

### ACP distribution in *Glossina* species

Distribution of ACP orthologs varies widely between species ( Figure 1). *Glossina* species have a disproportionately large number of α2-macroglobublin and heat shock proteins, which are important in immunity in *A. gambiae* and *Glossina* species ^26– 29^. Our analysis did not detect β-defensin orthologs, which are antimicrobial peptides involved in immune responses ^30^, in the *G. pallidipes* and *G. brevipalpis* genomes. We also did not detect Acp29AB, Acp70A, and andropin orthologs in this comprehensive catalogue of *Glossina* genes. This raises the possibility that these genes were lost by tsetse flies after evolutionary radiation of insects into multiple taxa, and alternative species-specific proteins might compensate for the same roles.

## Discussion

We performed comparative genomics analysis to detect the presence of male accessory gland proteins (ACPs) orthologs previously identified in *A. gambiae* and *Drosophila* ^1^. The motivation here was to improve knowledge on the biology of *Glossina* species ACPs given the importance of reproductive molecules in strategic designs of vector control. We identified 21 functional classes of ACP orthologs with secretory signals in five *Glossina* species genomes. We observed genetic signatures of a high rate of ACP protein divergence, supporting similar findings on male reproduction-related genes in *Drosophila* ^16^. ACPs exhibit high evolutionary changes, thus displaying between species divergence and within species polymorphism ^3, 31^. We restricted analysis to reference genes present in *A. gambiae* and *D. melanogaster*, and future studies integrating datasets from more closely related taxa will be useful to understand evolutionary changes associated with ACPs in more detail. α2-macroglobulins and heat shock proteins are the most abundant ACP orthologs in *Glossina* species. α2-macroglobulins are important in mosquito and *Drosophila* immunity ^26, 27^, and display signatures of purifying selection, suggesting they are critical for successful reproduction and all deleterious variations are purged. α2-macroglobulin over-representation, and the absence of other ACP orthologs implicated in immunity in the *Glossina* genomes points to their critical role in ensuring successful tsetse fly reproduction. Heat shock protein silencing in *A. gambiae* down-regulates up to 50% of male accessory gland proteins, half of which are male reproductive tract specific and encode the homologs of 13 known *Drosophila* ACPs that include Acp70A ^1^. Interestingly, *Glossina* species lack orthologs to Acp70A, andropin, Acp26Ab, Acp29AB, and Acp62F, which play critical roles in successful reproduction in *Anopheles* and *Drosophila* species ^1, 24, 25^.

Acp70A or sex peptide stimulates long-term post mating behavior, resulting in non-receptivity to mating and increased oviposition ^1, 25, 32^. Andropin is an antimicrobial peptide transferred to the female during copulation, and defends the female reproductive tract against microbes ^33^. Andropin also protects the male ejaculate from Gram-positive and Gram-negative bacterial infections ^1^. Acp26Ab stimulates oviposition in *Drosophila melanogaster* females ^34^, and together with Acp26Ab protects the male ejaculate from microbial infections, and displacement by a second ejaculate ^1^. *Drosophila* Acp29AB and Acp62F up-regulate genes for egg production and muscle development, although Acp29AB or Acp62F null males do not show a reproduction impairment phenotype ^35^. Acp62F also protects sperm in the female reproductive tract from protease attack ^36^.

A limitation in our study was the absence of transcriptome data to measure ACP differential gene expression. Analysis of transcriptome data available to others using a different approach (reciprocal BLASTp hits) identified only 4 non-annotated ACPs (GMOY000024, GMOY007757, GMOY009744, GMOY012189) orthologous to *D. melanogaster* not detected in our analysis; and no orthologs to *A. gambiae* ACPs ^37^. Future studies focused on transcriptome datasets of male accessory glands and testis may identify rapidly evolving *Glossina*-specific ACPs, which perhaps do not bear a well-known protein domains, and could escape *in silico* investigation dependent on a comparative genomic approach alone.

Our analysis detected orthologous *Glossina, Anopheles* and *Drosophila* ACPs belonging to the same functional classes, suggesting a conserved role for these proteins across all three genera. However, some ACPs may represent lineage-specific ACPs that may have evolved to perform species-specific reproductive functions. Our findings support evolutionary adaptation to different reproductive styles. Tsetse fly females produce a single fully developed larva at a time that pupates within minutes of exiting their uterus, and may have lost non-essential ACP genes after adaptation.

## Conclusions

Our findings provide genetic evidence of male accessory glad proteins in five recently sequenced *Glossina* genomes. It provides new avenues for molecular studies that evaluate potential field control strategies of these important vectors of human and animal disease.

## Data availability

The data referenced by this article are under copyright with the following copyright statement: Copyright: © 2017 Abry MF et al.

*Anopheles gambiae* and *Drosophila melanogaster* ACPs were obtained from: DOI, 10.1073/pnas.0703904104 ^1^.

*Glossina* species genome sequences were obtained from VectorBase.

## Supplementary material

**Supplementary Table 1:** (A) *Anopheles gambiae* (n=57) and *Drosophila melanogaster* (n=173) ACP orthologs. (B) ACP ortholog clusters identified in *Glossina*, *A. gambiae*, and *D. melanogaster* species prior to signal-peptide filtering.

**Supplementary File 1:** Amino acid sequences of the ACP ortholog clusters.

**Supplementary Figure 1:** Reconstructed phylogenies of the ACP orthologs within each cluster.
