# Supplementary material for: Comparative genomics identifies male accessory gland proteins in five Glossina species
Source: Wellcome Open Res. 2017 Nov 22;2:73. Originally published 2017 Aug 30. [Version 2] doi: 10.12688/wellcomeopenres.12445.2 (PMC5721568; doi:10.12688/wellcomeopenres.12445.2)
Supplement: Supplementary file 3 [file wellcomeopenres-2-14370-s0001.tgz › 62cf6f44-6fc5-4b77-aedc-60e63eaaa902.pdf]

α2-Macroglobulin: AGAP008364

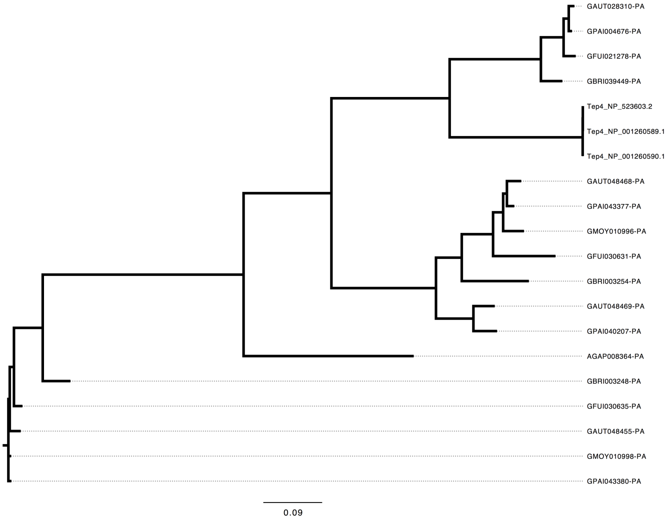

Cell adhesion: AGAP004428

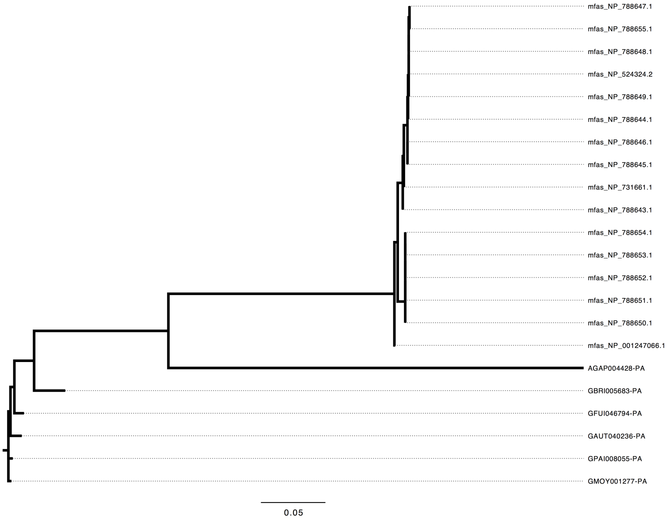

Carboxylesterase: AGAP005370

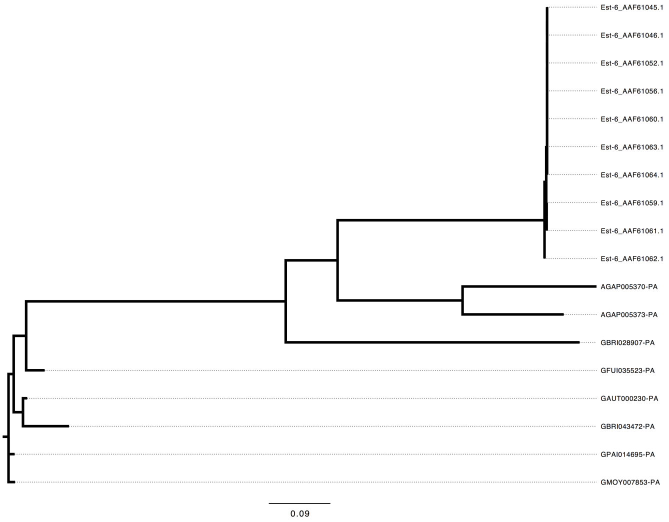

Chaperone: AGAP001424

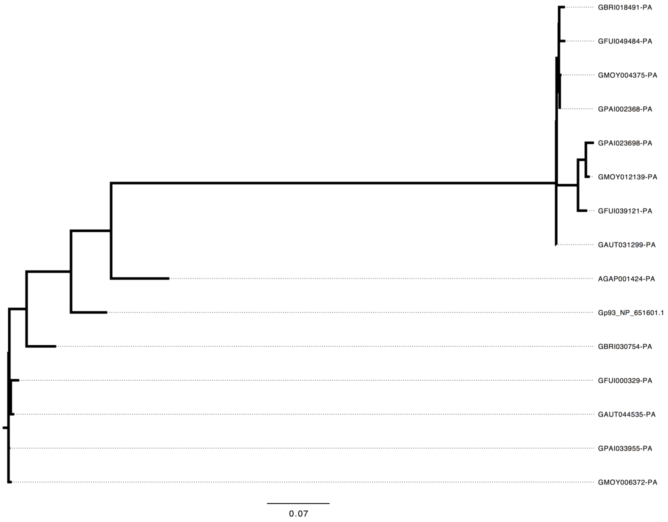

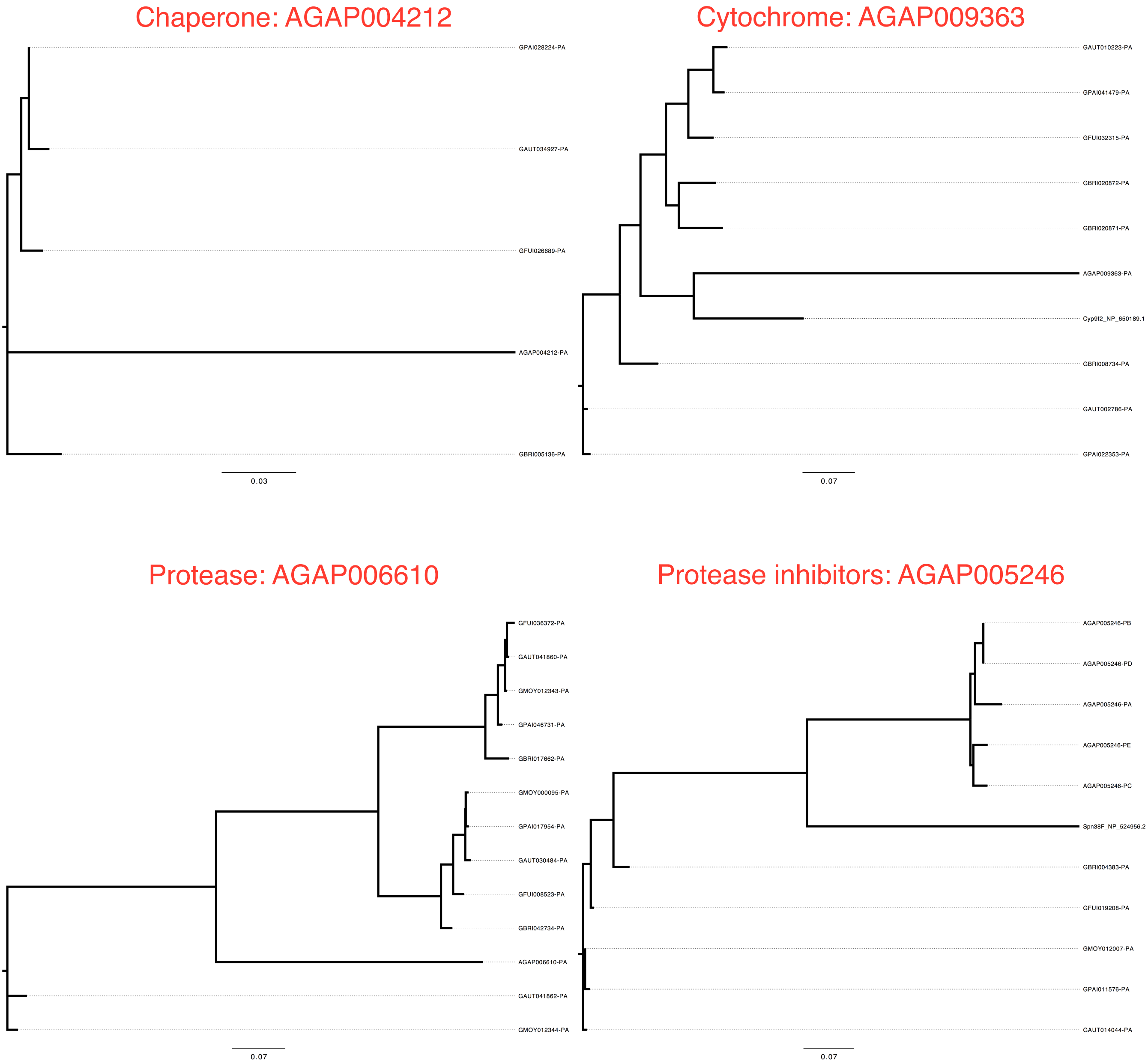

Chaperone: AGAP004212
GPAI028224-PA
GAUT034927-PA
GFUI026689-PA
AGAP004212-PA
GBRI005136-PA
0.03
Cytochrome: AGAP009363
GAUT010223-PA
GPAI041479-PA
GFUI032315-PA
GBRI020872-PA
GBRI020871-PA
AGAP009363-PA
Cyp9f2_NP_650189.1
GBRI008734-PA
GAUT002786-PA
GPAI022353-PA
0.07
Protease: AGAP006610
GFUI036372-PA
GAUT041860-PA
GMOY012343-PA
GPAI046731-PA
GBRI017662-PA
GMOY000095-PA
GPAI017954-PA
GAUT030484-PA
GFUI008523-PA
GBRI042734-PA
AGAP006610-PA
GAUT041862-PA
GMOY012344-PA
0.07
Protease inhibitors: AGAP005246
AGAP005246-PB
AGAP005246-PD
AGAP005246-PA
AGAP005246-PE
AGAP005246-PC
Spn38F_NP_524956.2
GBRI004383-PA
GFUI019208-PA
GMOY012007-PA
GPAI011576-PA
GAUT014044-PA
0.07

Protease inhibitors: Serpin-9

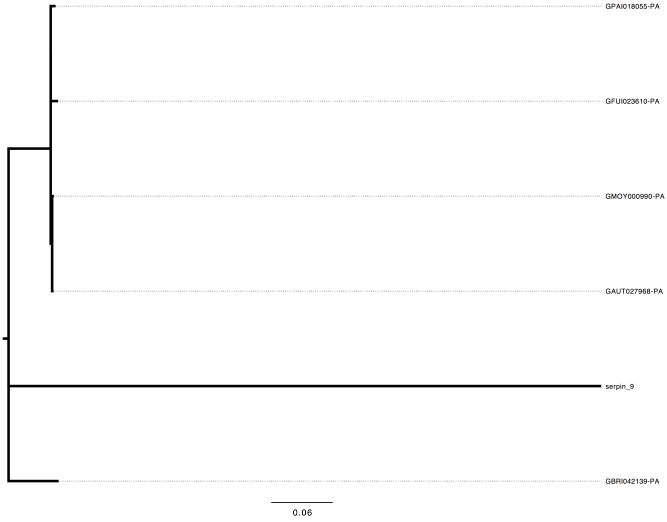

Protease inhibitors: Acp62F

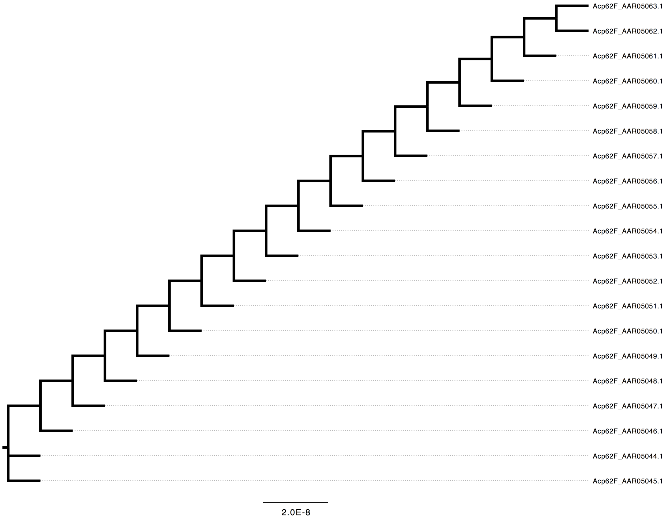

Protease inhibitors: Acp63F

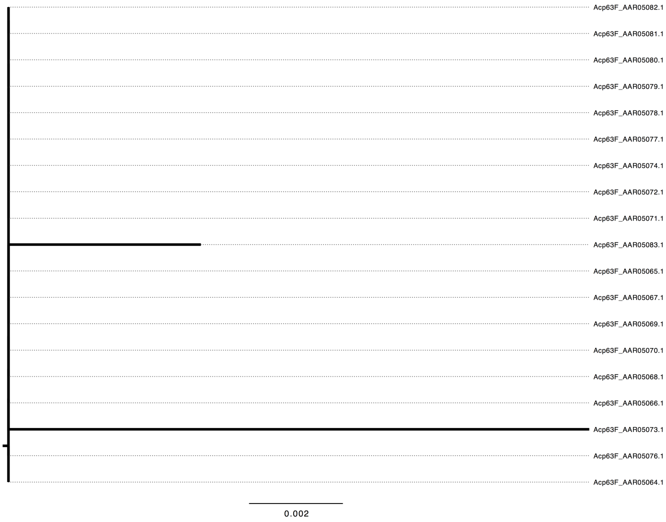

Protease inhibitors: AGAP006581

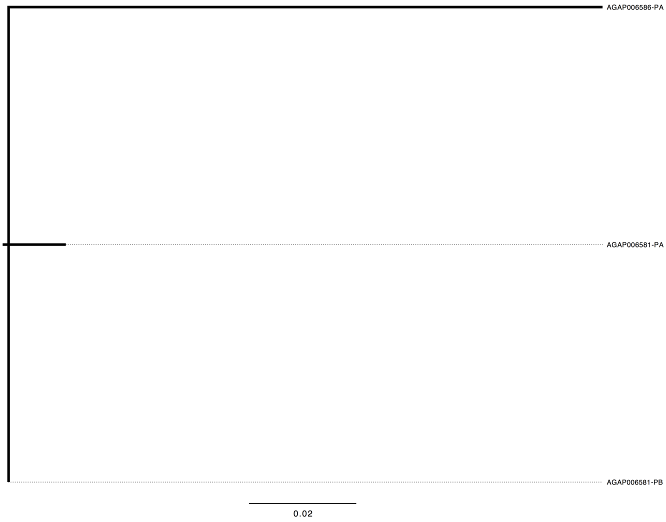

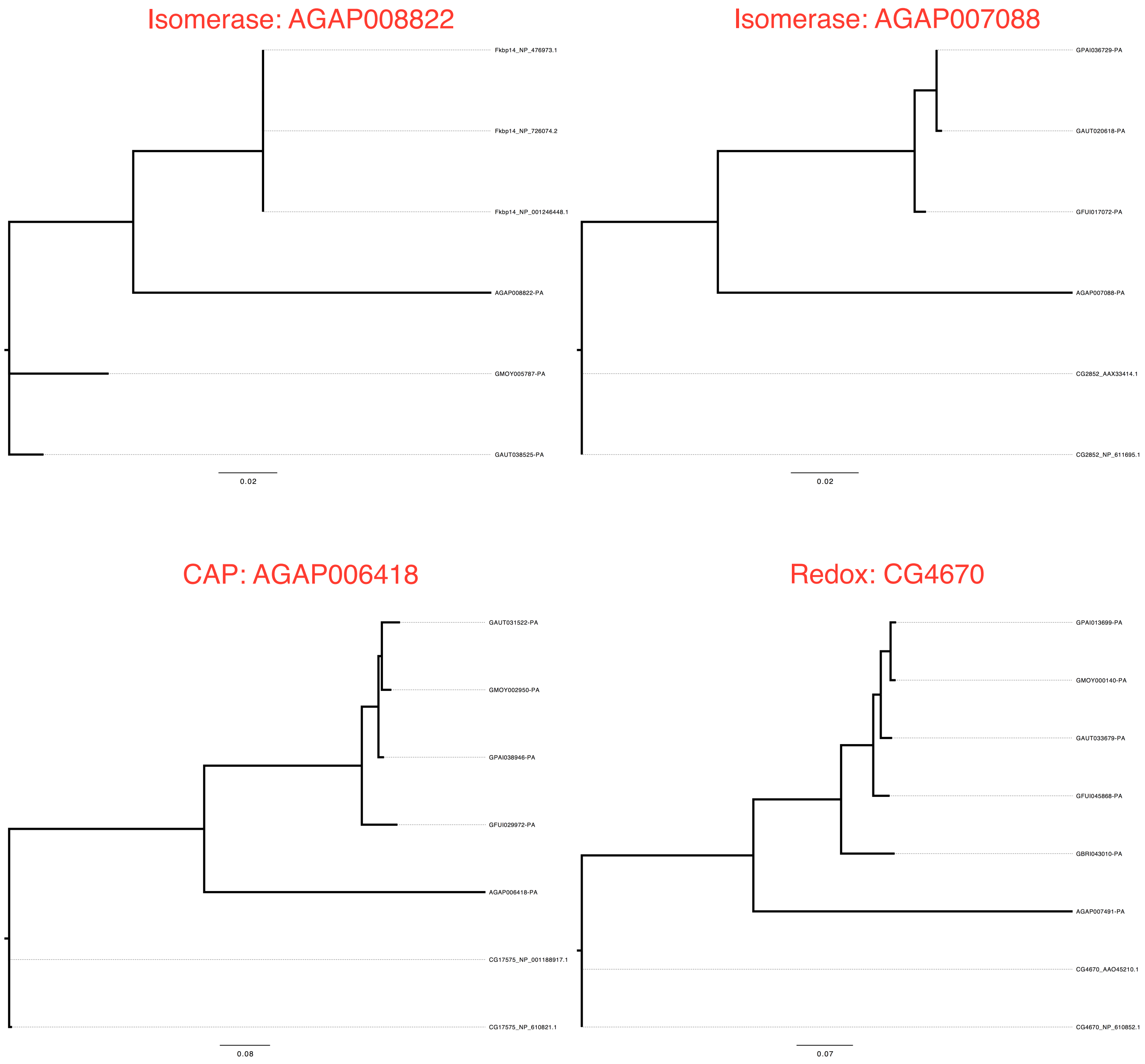

Isomerase: AGAP008822
Fkbp14_NP_476973.1
Fkbp14_NP_726074.2
Fkbp14_NP_001246448.1
AGAP008822-PA
GMOY005787-PA
GAUT038525-PA
0.02
Isomerase: AGAP007088
GPAI036729-PA
GAUT020618-PA
GFUI017072-PA
AGAP007088-PA
CG2852_AAX33414.1
CG2852_NP_611695.1
0.02
CAP: AGAP006418
GAUT031522-PA
GMOY002950-PA
GPAI038946-PA
GFUI029972-PA
AGAP006418-PA
CG17575_NP_001188917.1
CG17575_NP_610821.1
0.08
Redox: CG4670
GPAI013699-PA
GMOY000140-PA
GAUT033679-PA
GFUI045868-PA
GBRI043010-PA
AGAP007491-PA
CG4670_AAO45210.1
CG4670_NP_610852.1
0.07

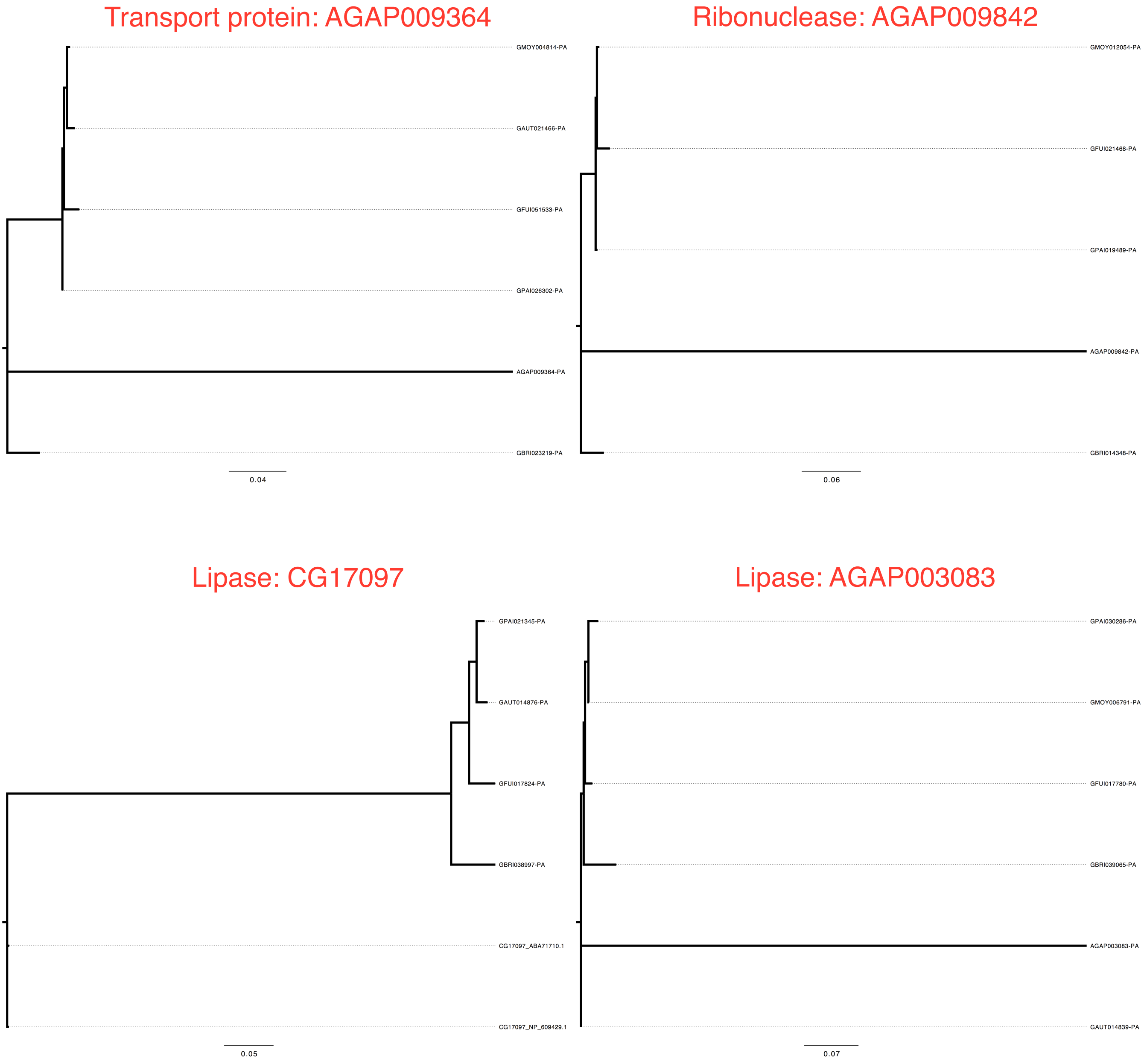
Transport protein: AGAP009364
GMOY004814-PA
GAUT021466-PA
GFUI051533-PA
GPAI026302-PA
AGAP009364-PA
GBRI023219-PA
0.04
Ribonuclease: AGAP009842
GMOY012054-PA
GFUI021468-PA
GPAI019489-PA
AGAP009842-PA
GBRI014348-PA
0.06
Lipase: CG17097
GPAI021345-PA
GAUT014876-PA
GFUI017824-PA
GBRI038997-PA
CG17097_ABA71710.1
CG17097_NP_609429.1
0.05
Lipase: AGAP003083
GPAI030286-PA
GMOY006791-PA
GFUI017780-PA
GBRI039065-PA
AGAP003083-PA
GAUT014839-PA
0.07

Calnexin: AGAP005032

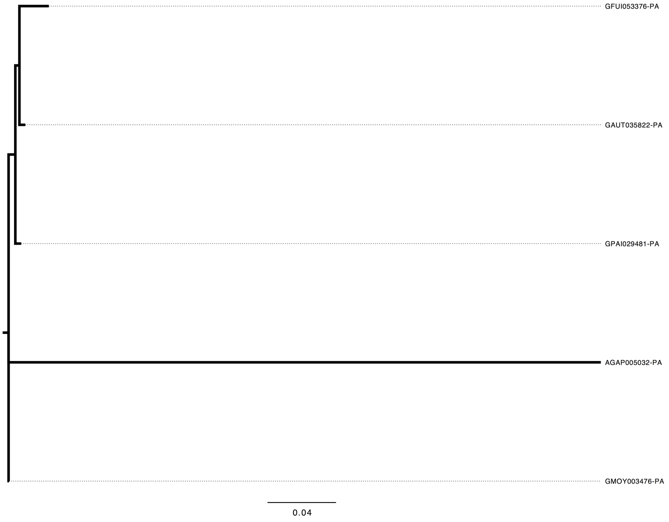

Beta-Defensin: AGAP007049

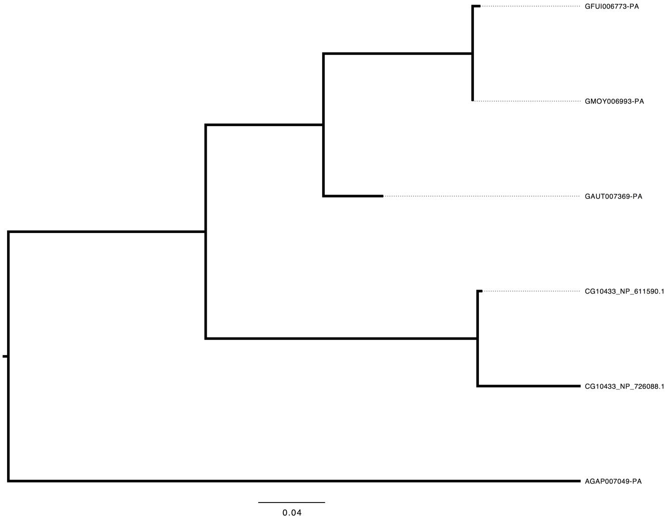

Accessory gland protein: CG14770

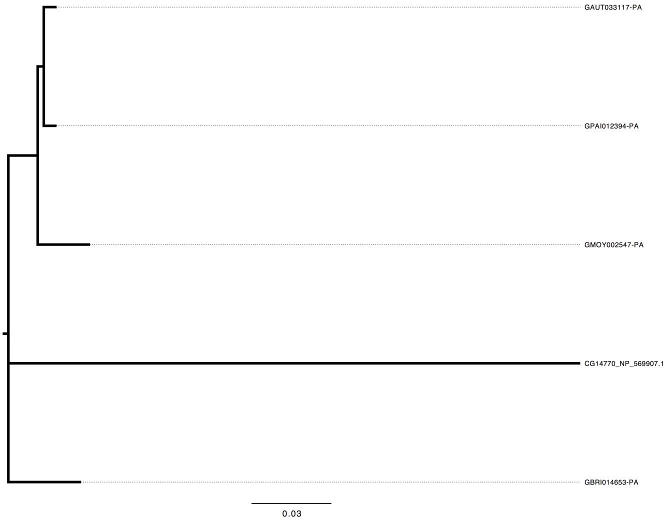

Accessory gland protein: Acp29AB

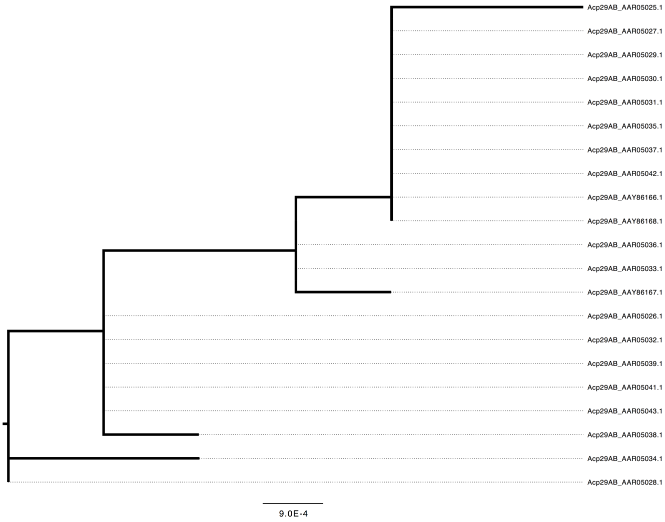

Accessory gland protein: Acp26Aa

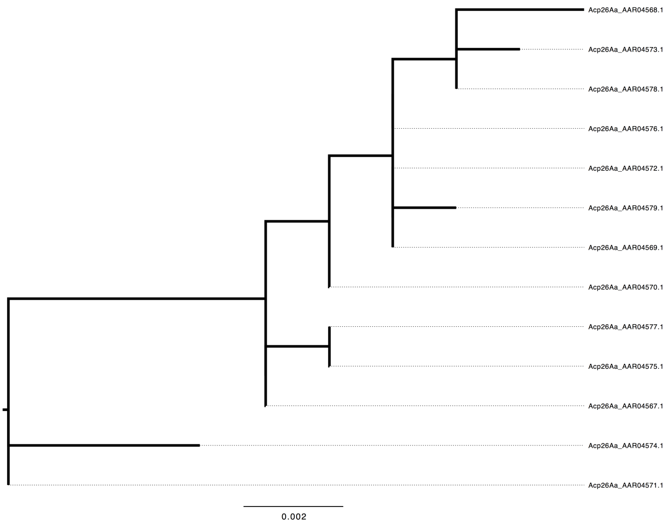

Accessory gland protein: Acp53Ea

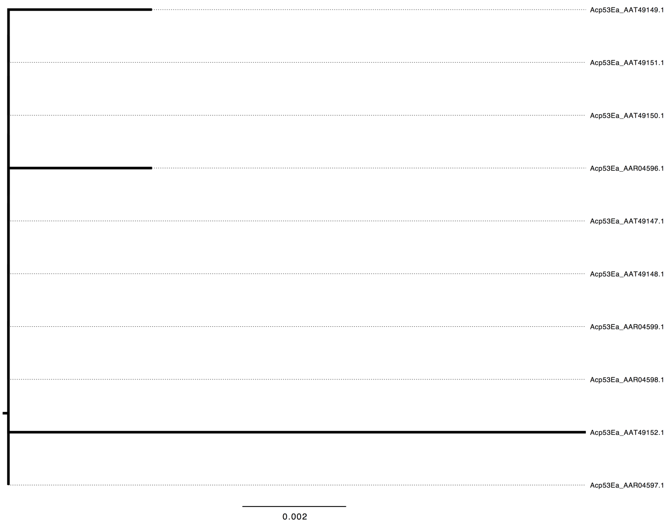

Accessory gland protein: AGAP009354

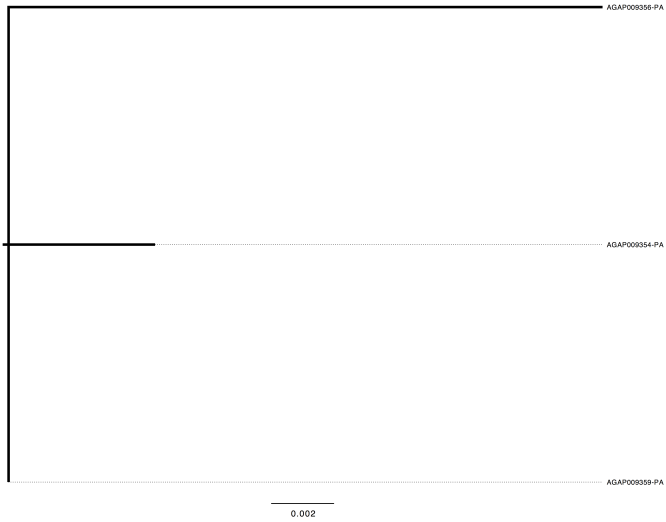

Heat shock protein: AGAP004192

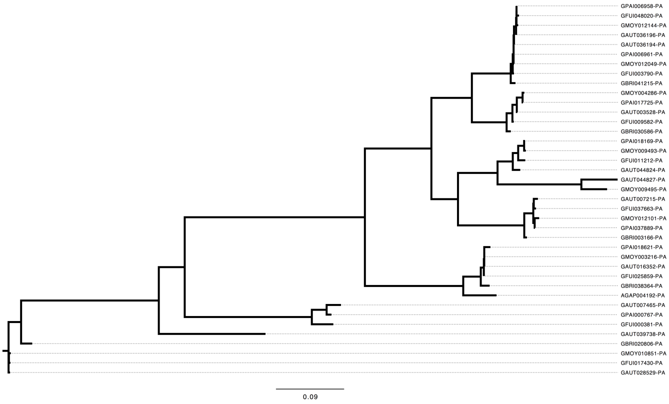

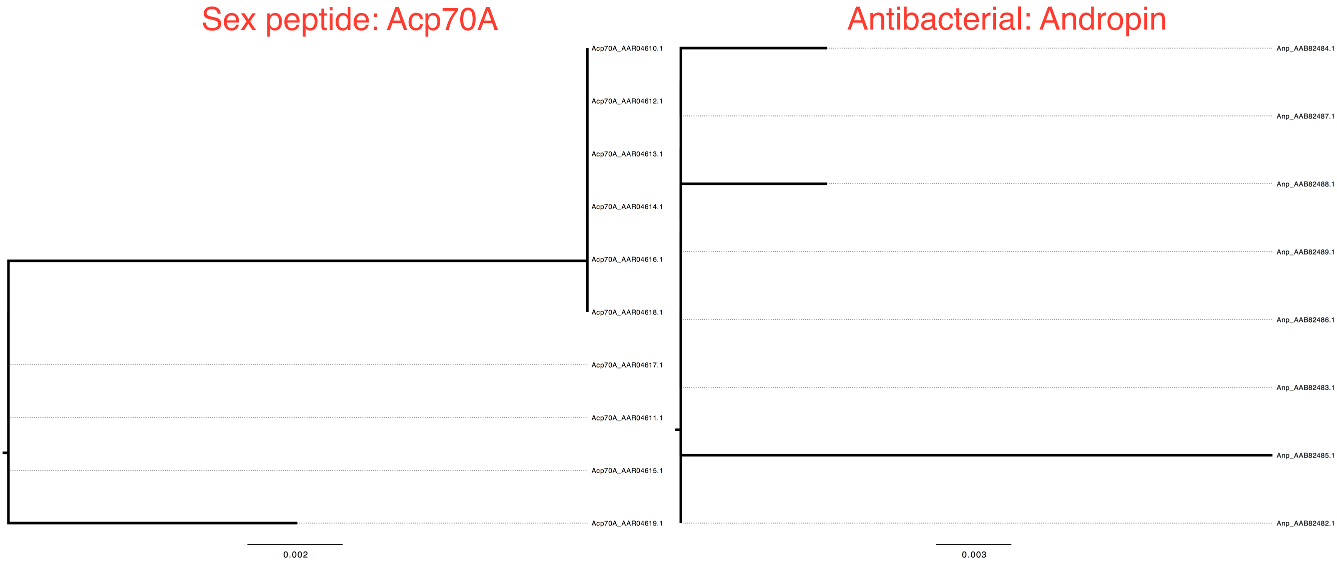
Sex peptide: Acp70A
Acp70A_AAR04610.1
Acp70A_AAR04612.1
Acp70A_AAR04613.1
Acp70A_AAR04614.1
Acp70A_AAR04616.1
Acp70A_AAR04618.1
Acp70A_AAR04617.1
Acp70A_AAR04611.1
Acp70A_AAR04615.1
Acp70A_AAR04619.1
0.002
Antibacterial: Andropin
Anp_AAB82484.1
Anp_AAB82487.1
Anp_AAB82488.1
Anp_AAB82489.1
Anp_AAB82486.1
Anp_AAB82483.1
Anp_AAB82485.1
Anp_AAB82482.1
0.003
