# Supplementary material for: Comparative genomics identifies male accessory gland proteins in five Glossina species
Source: Wellcome Open Res. 2017 Nov 22;2:73. Originally published 2017 Aug 30. [Version 2] doi: 10.12688/wellcomeopenres.12445.2 (PMC5721568; doi:10.12688/wellcomeopenres.12445.2)
Supplement: Peer review history

# Peer review: Referee response for version 2

**DOI:** 10.21956/wellcomeopenres.14370.r28295

**Published:** 2017-12-06

**Authors:** Kristipati Ravi Ram

**Affiliations:** 1 Embryotoxicology Laboratory, Environmental Toxicology Group, Council of Scientific & Industrial Research (CSIR)- Indian Institute of Toxicology Research, Lucknow, Uttar Pradesh, India

Authors have adequately addressed the concerns raised on the previous version.

I have read this submission. I believe that I have an appropriate level of expertise to confirm that it is of an acceptable scientific standard.

# Peer review: Referee response for version 1

**DOI:** 10.21956/wellcomeopenres.13476.r26501

**Published:** 2017-10-23

**Authors:** Kristipati Ravi Ram

**Affiliations:** 1 Embryotoxicology Laboratory, Environmental Toxicology Group, Council of Scientific & Industrial Research (CSIR)- Indian Institute of Toxicology Research, Lucknow, Uttar Pradesh, India

The paper by Abry *et al*., is aimed at identification of accessory gland proteins (Acps) in five *Glossina* species through bioinformatic approaches. Given the importance of reproductive molecules in strategic designs of vector control, the reported data are of significant value. However, the study is quite preliminary, and hence it feels that authors have gone overboard with the interpretation of these preliminary results. Primarily, authors are required to be clear about the rationale of their study given that the testicular/MAG contributions towards spermatophore formation through transcriptomic and proteomic approaches in *G. morsitans* has already been reported earlier (Scolari *et al.* 2016, Scientific Reports). The inclusion of data on reciprocal hits would increase the reliability of predicted/putative Acps. Further, RT-PCR based analysis of the enrichment/expression of at least a couple of identified putative Acp orthologs in male accessory glands (MAGs) from any of the Glossina species would add excellent value to the data and support to the conclusions drawn. Alternatively, authors may compare their datasets with RNAseq/proteomic data of *G. morsitans* (Scolari *et al.*, 2016) to assess if the identified genes are enriched/expressed in MAGs or form the part of male contributions to the spermatophore.

I have read this submission. I believe that I have an appropriate level of expertise to confirm that it is of an acceptable scientific standard, however I have significant reservations, as outlined above.

# Response

**Published:** 2017-11-20

**Authors:** Benard Kulohoma

We thank the reviewers for the comments. Our study is a preliminary *in silico* exploration of the MAG repertoire of *Glossina* species, and provides initial findings about genome annotations that identifies ACPs orthologs. Our aim was to establish whether there are male accessory gland (ACPs) orthologs across the Glossina species genomes, by comparing them to well characterised ACP orthologs from *Drosophila melanogaster* and *Anopheles gambiae*, given the importance of reproductive molecules in strategic designs of vector control.

 

- We have revised the manuscript to address the over-emphasis that ACPS have a play crucial role in the tsetse fly life reproductive cycle, and thus vector control. Lines 45, 73, 163, and 164.
- We have revised the manuscript to show that this is a preliminary study using the initial release of the Glossina genomes, which are publicly available, and future studies using RNASeq/transcriptome datasets of the male accessory glands and testis may identify rapidly evolving Glossina-specific ACPs, which perhaps do not bear a well-known protein domains, and could escape *in silico* investigation dependent on a comparative genomic approach alone. Lines 194 - 201.
- We have amended the manuscript to reflect that we identified possible signatures of selection, since a wider analysis including more closely related taxa was not performed. Lines 137, 140-143, and 165-166.

# Peer review: Referee response for version 1

**DOI:** 10.21956/wellcomeopenres.13476.r25531

**Published:** 2017-09-04

**Authors:** Emiliano Mancini

**Affiliations:** 1 Department of Science, Roma Tre University, Rome, Italy

The paper by Abry *et al.* presents a comparative genomics analysis to identify male accessory gland proteins in *Glossina *species. The paper is an " *in silico*" exploration of the MAG repertoire of *Glossina*, and has the merit to open the road to further investigation on these important reproductive proteins and their functions. However I think that the authors should give the right relevance to their results, which are, in a sense, quite preliminary, but presented, in my opinion, with too much emphasis.

 First of all the authors have to clarify the aim of the work, especially in the context of vector control. Although it is true that a deep understanding of ACPS in *Glossina* can be useful for limit fertility of *Glossina* species and thus control their spread (these proteins have important roles in female post-mating behaviour, such as single mating, storing and sustaining viability of sperms etc.), I think that ACPS do not necessarily have a realistic connection with the peculiar post-mating characteristic of the reproductive cycle of *Glossina *(e.g. absence of eggs deposition). Since this point is recurrently discussed and emphasized in the paper, I suggest the author either to better explain how the ACPS can have a role on the origin of this particular life cycle, or to eliminate this point which, otherwise, remains quite speculative.

 It is important that authors stress the importance that their work should be completed in the future by RNAseq of male accessory glands and testis of *Glossina*. This because some rapidly evolving ACPS could escape *in silico *investigation, as they could be *Glossina*-specific and not corresponding to any ortholog, or simply because they do not bear a well-known protein domain. Species-specific proteins can not be obtained by a comparative genomic approach alone, so that RNA analysis is required to reveal (probably) the most important, peculiar ACPS in this important vector.

 Finally, I would be very cautious in assigning positive selection to genes with dN/dS>1, since a wider analysis including more closely related taxa (and data on their intra-specific variability) should be performed. However, based on the dN/dS estimation here provided, I think that the authors should just argue about "possible signatures of positive selection" on these genes.

I have read this submission. I believe that I have an appropriate level of expertise to confirm that it is of an acceptable scientific standard.

# Response

**Published:** 2017-11-20

**Authors:** Benard Kulohoma

We thank the reviewers for the comments. Our study is a preliminary *in silico* exploration of the MAG repertoire of *Glossina* species, and provides initial findings about genome annotations that identifies ACPs orthologs. Our aim was to establish whether there are male accessory gland (ACPs) orthologs across the Glossina species genomes, by comparing them to well characterised ACP orthologs from *Drosophila melanogaster* and *Anopheles gambiae*, given the importance of reproductive molecules in strategic designs of vector control.

 

- We have revised the manuscript to address the over-emphasis that ACPS have a play crucial role in the tsetse fly life reproductive cycle, and thus vector control. Lines 45, 73, 163, and 164.
- We have revised the manuscript to show that this is a preliminary study using the initial release of the Glossina genomes, which are publicly available, and future studies using RNASeq/transcriptome datasets of the male accessory glands and testis may identify rapidly evolving Glossina-specific ACPs, which perhaps do not bear a well-known protein domains, and could escape *in silico *investigation dependent on a comparative genomic approach alone. Lines 194 - 201.
- We have amended the manuscript to reflect that we identified possible signatures of selection, since a wider analysis including more closely related taxa was not performed. Lines 137, 140-143, and 165-166.
